# RETRACTION: Evaluation of Risk Factors for Surgical Site Infections in Osteoarthritis Patients Undergoing Total Knee Arthroplasty

**DOI:** 10.1111/iwj.70594

**Published:** 2025-04-21

## Abstract

**RETRACTION:** LiH. , LiY. , WangD. , HuangQ. , and LiuD. , “Evaluation of Risk Factors for Surgical Site Infections in Osteoarthritis Patients Undergoing Total Knee Arthroplasty,” International Wound Journal 21, no. 3 (2024): e14521, 10.1111/iwj.14521.37997562 PMC10898376

The above article, published online on 23 November 2023, in Wiley Online Library (http://onlinelibrary.wiley.com/), has been retracted by agreement between the journal Editor in Chief, Professor Keith Harding; and John Wiley & Sons Ltd. Following an investigation by the publisher, all parties have concluded that this article was accepted solely on the basis of a compromised peer review process. The editors have therefore decided to retract the article. The authors did not respond to our notice regarding the retraction.

**RETRACTION:** H. Li , Y. Li , D. Wang , Q. Huang , and D. Liu , “Evaluation of Risk Factors for Surgical Site Infections in Osteoarthritis Patients Undergoing Total Knee Arthroplasty,” International Wound Journal 21, no. 3 (2024): e14521, 10.1111/iwj.14521.37997562 PMC10898376

The above article, published online on 23 November 2023, in Wiley Online Library (http://onlinelibrary.wiley.com/), has been retracted by agreement between the journal Editor in Chief, Professor Keith Harding; and John Wiley & Sons Ltd. Following an investigation by the publisher, all parties have concluded that this article was accepted solely on the basis of a compromised peer review process. The editors have therefore decided to retract the article. The authors did not respond to our notice regarding the retraction.

